# Label-free quantitative proteomic analysis of ethanamizuril-resistant versus -sensitive strains of *Eimeria tenella*

**DOI:** 10.1186/s13071-022-05412-6

**Published:** 2022-09-08

**Authors:** Peipei Cheng, Chunmei Wang, Lifang Zhang, Chenzhong Fei, Yingchun Liu, Mi Wang, Keyu Zhang, Xiaoyang Wang, Feng Gu, Feiqun Xue

**Affiliations:** Key Laboratory of Veterinary Chemical Drugs and Pharmaceutics, Ministry of Agriculture and Rural Affairs/Shanghai Veterinary Research Institute, Chinese Academy of Agricultural Sciences, 518 Ziyue Road, Minhang District, Shanghai, 200241 China

**Keywords:** Anticoccidial, Drug resistance, *Eimeria tenella*, Ethanamizuril, Label-free proteomics

## Abstract

**Background:**

Avian coccidiosis is an important parasitic disease that has serious adverse effects on the global poultry industry. The extensive use of anticoccidial drugs has resulted in an increase in drug resistance. Ethanamizuril (EZL) is a novel triazine with high anticoccidial activity.

**Methods:**

We compared oocyst production and sporulation between EZL-sensitive (S) and EZL-resistant *Eimeria tenella* strains (R10 and R200) and used label-free quantitative proteomics to identify differentially expressed proteins (DEPs) between these strains.

**Results:**

We generated two EZL-resistant *E. tenella* strains: strain R10, which was induced using a constant dose of 10 mg EZL/kg poultry feed, and strain R200, which was generated by gradually increasing the EZL dosage to 200 mg EZL/kg poultry feed. With an increase in resistance, the total oocyst output decreased, but the percentage of sporulation did not change significantly. We identified a total of 7511 peptides and 1282 proteins, and found 152 DEPs in the R10 strain versus the S strain, 426 DEPs in the R200 strain versus the S strain and 494 DEPs in the R200 strain versus the R10 strain. When compared with the S strain, 86 DEPs were found to have consistent trends in both resistant strains. The DEPs were primarily involved in ATP and GTP binding, invasion, and membrane components. Gene Ontology and Kyoto Encyclopedia of Genes and Genomes pathway analyses of the DEPs suggested that they are involved in transcription and translation processes. Protein–protein interaction network analysis of the 86 DEPs showed that 10 proteins were hubs in the functional interaction network (≥ 8 edges) and five of them were ribosomal proteins.

**Conclusions:**

The results of the present study indicate that the resistance mechanisms of *E. tenella* against EZL might be related to the transcriptional and translational processes, especially in the factors that inhibit the growth of parasites. The DEPs found in this study provide new insights into the resistance mechanisms of *E. tenella* against EZL. Further research on these potential targets holds promise for new chemotherapeutic approaches for controlling *E. tenella* infections.

**Graphical Abstract:**

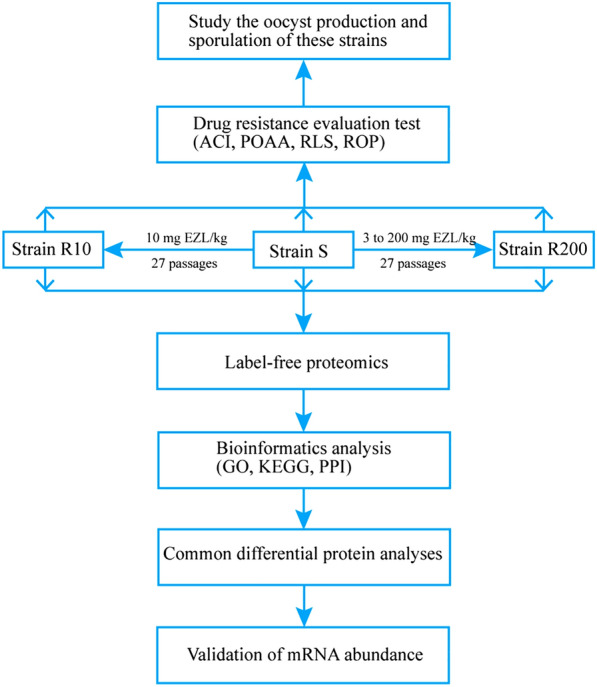

**Supplementary Information:**

The online version contains supplementary material available at 10.1186/s13071-022-05412-6.

## Background

Avian coccidiosis is an infectious intestinal parasitic disease that severely impairs the growth and development of chickens, thus adversely affecting the global poultry industry, with losses that exceed £10.36 billion per year [1]. Disease is spread via the oral-fecal route through the ingestion of sporulated oocysts of *Eimeria* spp. Anticoccidial drugs are often used to treat coccidiosis, but the persistent use of these drugs has led to microbial resistance [2], which driven the continuous search for and development of new anticoccidial drugs. Ethanamizuril (EZL) is a novel triazine anticoccidial compound, developed at the Shanghai Veterinary Research Institute, Chinese Academy of Agriculture Sciences. This drug is safe and possesses high anticoccidial activity with an anticoccidial index (ACI) > 180 [3]. The recommended dose is 10 mg/kg in poultry feed. As prophylactic chemotherapy with anticoccidial drugs is the primary method to control coccidiosis, this drug may have an enormous potential for future applications. However, limited research has been carried out on EZL, and no studies so far have explored the ability of *Eimeria* spp. to develop resistance against it.

*Eimeria tenella* is one of the seven species that cause coccidiosis in chickens [4]. Isotopic labeling, with tandem mass tags and amino acids in cell culture, has been successfully applied to study the resistance mechanisms of *E. tenella* to monensin [5, 6]. Ma et al. also used phosphoproteomics to compare the life-cycle stages of four *E. tenella* strains [7]. Additionally, label-free quantification (LFQ) proteomics has been used to explore the mechanisms of action of nitromezuril and EZL on second-generation merozoites of *E. tenella* [8].

Label-free proteomics has a greater detection capacity and pathway integration capability than other quantitative methods. Therefore, in this study, we investigated the resistance mechanisms of *E. tenella* to EZL using this label-free proteomic approach on sporulated oocysts. We also compared the proteomic profiles of EZL-sensitive and EZL-resistant strains.

## Methods

### Parasites

An EZL-sensitive *E. tenella* (S) strain, without any anticoccidial treatment, was obtained from the Key Laboratory of Animal Parasitology of the Ministry of Agriculture. We then generated two EZL-resistant *E. tenella* strains (R10 and R200) by inoculating 2-week-old chickens, which were given feed containing EZL, with 27 in vivo passages of oocysts from strain S. Chickens were administered via oral gavage a single dose of 8 × 10^4^ *E. tenella* sporulated oocysts from the three different strains. Three groups (10 chickens/group) were passaged: group S, the sensitive strain with EZL-free feed; group R10, a resistant strain induced by a constant dose of 10 mg EZL/kg (poultry feed); and group R200, a resistant strain induced by gradually increasing the EZL dosage from 3 to 200 mg EZL/kg. The schedule for gradually increasing the EZL dosage from 3 to 200 mg/kg in the chicken feed was as follows: 3 mg/kg, two passages; 5 mg/kg, one passage; 10 mg/kg, seven passages; 20 mg/kg, two passages; 40 mg/kg, two passages; 60 mg/kg, one passage; 80 mg/kg, two passages; 100 mg/kg, two passages; 120 mg/kg, two passages; and 200 mg/kg, six passages. Additional file 1: Table S1 shows the schedule for increasing the EZL dosage from 3 to 200 mg/kg chicken feed. When the oocyst production in group R200 was similar to that in group S (the same order of magnitude), we increased the dosage of EZL. A single oocyst was isolated at the dosage of 10 mg/kg during the process of inducing the R200 strain. The single oocyst of each of the three strains was identified using PCR method [9] (Additional file 1: Fig. S1).

Unsporulated oocysts were collected from the chicken cecum of all the groups and incubated at 28 °C in 2.5% potassium dichromate until > 80% had sporulated, following which they were purified using the sodium hypochlorite method [10]. Purified samples that were > 90% sporulated were used for label-free quantitative proteomic with three biological replicates.

### Chickens

One-day-old chickens were purchased from a local hatchery (Min You, Shanghai, China). Chickens were caged with ad libitum access to water and drug-free feed for 2 weeks before being used in the experiments.

This study was conducted under the guidance of the Institutional Animal Care and Use Committee of China and was approved by the Ethics Committee of the Shanghai Veterinary Research Institute (Shvriro-2014120884).

### Drug resistance evaluation test

The resistance of the strains was measured using a drug resistance evaluation test. In this experiment, 300 2-week-old chickens were divided into 10 groups, with 30 chickens in each group (10 chickens per cage × 3 replications). These 10 groups included: the uninfected-unmedicated control group (NNC); three infected-unmedicated experimental groups (infected with strains S, R10 and R200) and six infected-medicated experimental groups (infected with strain S and medicated with 10 and 200 mg EZL/kg; infected with strain R10 and medicated with 10 and 200 mg EZL/kg; infected with strain R200 and medicated with 10 and 200 mg EZL/kg). Additional file 1: Table S2 shows how these 10 groups were characterized. We evaluated drug sensitivity using four indices: the ACI, percentage of optimum anticoccidial activity (POAA), reduction in lesion scores (RLS) and relative oocyst production (ROP). These indices were calculated according to Lan et al. [11], as shown in Additional file 1: Supplementary material S1.Scores were presented as the mean ± standard deviation (SD). The strain was judged to be resistant at ACI < 160, and to be sensitive at ACI ≥ 160. A strain with a POAA and RLS < 50% was judged to be resistant, and a strain with POAA and RLS > 50% was judged to be sensitive. Strains with an ROP > 15% and < 15% were judged to be resistant and sensitive, respectively. When all four indices indicated resistance, the strain was evaluated as being completely resistant; if three of the four indices indicated resistance, the strain was considered to be highly resistant; moderate resistance referred to strains with two of the four indices indicating resistance; and when only one of the four indices indicated resistance, the strains were considered to show slight resistance [3].

### Comparing the oocyst production and sporulation of the strains

A total of 90 14-day-old chickens were divided into three groups of 30 chickens each. Each of the three groups was further subdivided into three cages with 10 chickens in one cage. The three groups consisted of the inoculated EZL-sensitive strain (S) and two resistance strains (R10, R200), respectively. An aliquot of 1000 *E. tenella* sporulated oocysts were inoculated into each chicken through gavage. Fecal oocysts were counted from days 5 to 12 post-infection. The total output of oocysts was calculated using a modified McMaster chamber [12].

Oocysts, at a concentration of 5 × 10^5^ oocysts/ml, were collected from the cecum of chickens in the three groups and incubated in 100 ml of 2.5% potassium dichromate solution for 48 h at 28 °C. Oocyst sporulation was counted at 24 and 48 h. Approximately 500 oocysts were counted at each time point, including both sporulated and unsporulated oocysts. The percentage of sporulation = the number of sporulated oocysts/the total number of oocysts × 100%.

### Protein preparation and digestion

Proteins were prepared from sporulated oocysts by suspending oocytes in 500 µl STD buffer (150 mM Tris–HCl pH 8.0 containing 4% sodium dodecyl sulfate and 1 mM dithiothreitol). The samples were boiled for 5 min and then sonicated on ice 10 times (80 W ultrasound at 10 s, then 15-s pause). This crude extract was boiled and clarified by centrifugation at 16,000 *g* at 25 °C for 10 min. The protein content of supernatants was determined with a BCA protein assay kit (Bio-Rad Laboratories, Hercules, CA, USA) and samples were stored at − 80 °C until use.

The crude protein extracts (200 μg) were digested using a published protocol [13]. Briefly, 200 μl UA buffer (8 M urea, 150 mM Tris–HCl pH 8.0) was added to the samples, followed by two ultrafiltration steps consisting of centrifugation at 14,000 *g* for 15 min using 10-kDa cutoff filter units (Pall Corp., Port Washington, NY, USA). Iodoacetamide (100 µl of 50 mM in UA buffer) was added to the sample in the filter unit and then incubated in the dark for 30 min. The filter was washed twice with 100 μl UA buffer and twice with 100 μl 100 mM NH_4_HCO_3_. Trypsin (4 μg) in 40 μl of 25 mM NH_4_HCO_3_ was added and the sample was incubated for 16–18 h at 37 °C. Digested proteins were recovered by centrifugation as described above. The filtrate was collected in a new tube and the peptide content was estimated by UV spectroscopy at 280 nm using an extinction coefficient of 1.1 of 0.1% (g/l) solution [14].

### Peptide identification

Peptides were desalted using solid-phase extraction cartridges (Empore C18-SD [standard density]; bed I.D.: 7 mm; volume: 3 ml; Sigma-Aldrich, St. Louis, MO, USA) and concentrated by vacuum centrifugation. Samples were then dissolved in 40 µl 0.1% (v/v) trifluoroacetic acid. Peptides were separated using a nanoliter liquid chromatography system (Easy nLC1000; Thermo Fisher Scientific, Waltham, MA, USA) and identified by mass spectrometry (MS) using a Q Exactive mass spectrometer (Thermo Fisher Scientific). Peptides (2 μg) were loaded onto a C18-reversed phase column (Thermo Fisher Scientific Easy Column, 10 cm × 75 μm, 3 μm) and separated with gradient conditions of buffer A (2% acetonitrile and 0.1% formic acid) and buffer B (84% acetonitrile and 0.1% formic acid) at a flow rate of 300 nl/min with run time of 120 min. The gradient conditions were as follows: 0 min, 100% A; 0–110 min, 0–45% B; 110–117 min, 45–100% B; 117–120 min, 100% B. MS data were acquired using a data-dependent top 20 method dynamically choosing the most abundant precursor ions from the survey scan (300–1800 *m*/*z*) for HCD fragmentation. Determination of the target value was based on the predictive automatic gain control that was set at 3e^6^. The instrument was run with peptide recognition mode enabled and adynamic exclusion duration of 40 s. The resolution of the survey scans and HCD spectra were set to 70,000 at* m*/*z* 200 and 17,500 at* m*/*z* 200, respectively. The maximum ion injection times of the MS1 and MS2 scan were 10 and 60 ms, respectively, and the normalized collision energy was 30 eV; the underfill ratio was defined as 0.1%. Each sample was analyzed in triplicate.

### Sequence database searching and data analysis

The MS data were analyzed using MaxQuant software (version 1.3.0.5) (www.biochem.mpg.de). MS data were searched against the Uniprot *Eimeria tenella* database. An initial search was set with a precursor mass window of 6 ppm. The search followed an enzymatic cleavage rule of trypsin KR/P and a mass tolerance of 20 ppm for fragment ions; a maximum of two missed cleavage sites were allowed. Fixed modification of carbamido methylation of cysteine and variable modifications of protein N-terminal acetylation and methionine oxidation were used for database searching. The peptide and protein identification were filtered with a false discovery rate of 0.01. Label-free quantification (LOQ) was carried out using the MaxQuant software as previously described [15, 16]. The decoy database pattern was set as reverse and LOQ min ratio count was set as 1. Protein abundance was calculated on the basis of the normalized spectral protein intensity (LFQ intensity).

The resultant MaxQuant data were analyzed using Perseus statistical software (version 1.3.0.5; www.biochem.mpg.de). Comparisons of each two-sample set were performed using the Student’s two-tailed t-test. A *P* value < 0.05 and ratio > 1.5 or < 0.667 were considered to indicate significant differences.

### Bioinformatics analysis

Proteins that were judged significantly different between comparison groups were subjected to Gene Ontology (GO; https://www.blast2go.com/) analysis using Blast2 GO to annotate differentially expressed proteins that were classified by cell component, biological process and molecular function. Kyoto Encyclopedia of Genes and Genomes (KEGG) pathway analysis (http://www.genome.jp/kegg/) was used for pathway analysis of DEPs. The interaction network of DEPs was analyzed using the STRING database (http://string-db.org/). We selected the medium confidence (0.4) as the minimum required interaction score.

### Quantitative real-time PCR analysis

Total RNA was extracted from sporulated oocysts using TRIzol reagent according to the manufacturer’s instructions (Invitrogen, Thermo Fisher Scientific, Carlsbad, CA, USA) and reverse transcribed with the Prime Script RT reagent kit with gDNA Eraser (Takara, Dalian, China). Quantitative PCR of complementary DNA was carried out using a QuantiNova SYBR Green PCR kit (Qiagen, Hilden, Germany) and custom primers (Additional file 1: Table S3). Amplification conditions were: 2 min at 95 °C, and 40 cycles of 95 °C for 5 s, 60 °C for 34 s. The *E. tenella* 18S ribosomal RNA (rRNA) was used as internal reference to normalize all quantitative real-time (qPCR) data. Relative expression levels were calculated using the 2^−△△Ct^ method [17].

### Statistical analysis

GraphPad Prism 8.0 was used to generate graphs (GraphPad Software, San Diego, CA, USA). All data were expressed as mean ± SD. Statistical differences were determined by two-tailed Student’s t-test between two groups. The criterion for statistical significance was *P < 0.05, **P < 0.01 and ***P < 0.001.

## Results

### Drug resistance trials

The infected groups treated with 10 mg EZL/kg (poultry feed) had the following mean ACI (± SD) scores: strain S, 199.43 ± 11.16; strain R10, 118.53 ± 14.55; and strain R200, 122.49 ± 4.90. When the dosage was 200 mg EZL/kg, the ACI scores were 195.74 ± 4.14, strain S; 180.3 ± 4.37, strain R10; and 89.14 ± 13.00, strain R200. These results showed that 10 mg EZL/kg was highly effective against strain S, but ineffective against strains R10 and R200. A dosage of 200 mg EZL/kg was effective against strains S and R10, but not against R200. Table 1 and Additional file 1: Table S4 shows the scores from the indices used to evaluate the resistance of *E. tenella* against EZL. These results suggest that strain S was sensitive to EZL, R10 was resistant to 10 mg EZL/kg but sensitive to 200 mg EZL/kg and R200 was completely resistant to EZL.

**Table 1:** Results of the drug resistance evaluation test on *Eimeria tenella* strains against ethanamizuril

Strains^a^	Dose (mg EZL/kg poulty feed)	ACI	POAA	RLS	ROP	Conclusion
S	10	199.43 ± 11.16 (−)	127.02 ± 21.06 (−)	60.71 ± 6.90 (−)	0.63 ± 0.56 (−)	Sensitive
	200	195.74 ± 4.14 (−)	84.58 ± 15.18 (−)	100 ± 0.00 (−)	0.00 ± 0.00 (−)	Sensitive
R10	10	118.53 ± 14.55 (+)	11.33 ± 17.33 (+)	8.89 ± 15.40 (+)	67.30 ± 4.25 (+)	Completely resistant
	200	180.3 ± 4.37 (−)	70.51 ± 2.31 (−)	86.69 ± 6.69 (−)	2.81 ± 0.67 (−)	Sensitive
R200	10	122.49 ± 4.90 (+)	0.52 ± 25.60 (+)	22.92 ± 3.61 (+)	116.39 ± 36.14 (+)	Completely resistant
	200	89.14 ± 13.00 (+)	− 124.04 ± 89.15 (+)	12.50 ± 6.25 (+)	93.41 ± 10.85 (+)	Completely resistant

Data are presented as the mean ± standard deviation (SD) unless indicated otherwise; (+) means EZL resistant; (−) means EZL sensitive

* EZL* Ethanamizuril, *ACI* Anticoccidial index, *POAA* percentage optimum anticoccidial activity, *RLS* reduction of lesion scores, *ROP* relative oocyst production

^a^S, EZL-sensitive strain, R10, R200 EZL-resistant strains

### Comparing the oocyst production and sporulation of the strains

The fecal oocyst output of the resistant strains (R10, R200) was lower than that of the sensitive strain (S) (Additional file 1: Fig. S2A). The total oocyst output of group R200 was significantly decreased compared to that of group S (Additional file 1: Fig. S2B). The percentage of sporulation in group R10 was not significantly different from that of group S at 24 and 48 h (Additional file 1: Fig. S3; Additional file 1: Table S5). At 24 h, the percentage of sporulation in group R200 was significantly different from that of group S; however, there was no significant difference at 48 h (Additional file 1: Fig. S3, Table S5).

### Protein identification and quantification

**Fig. 1:**
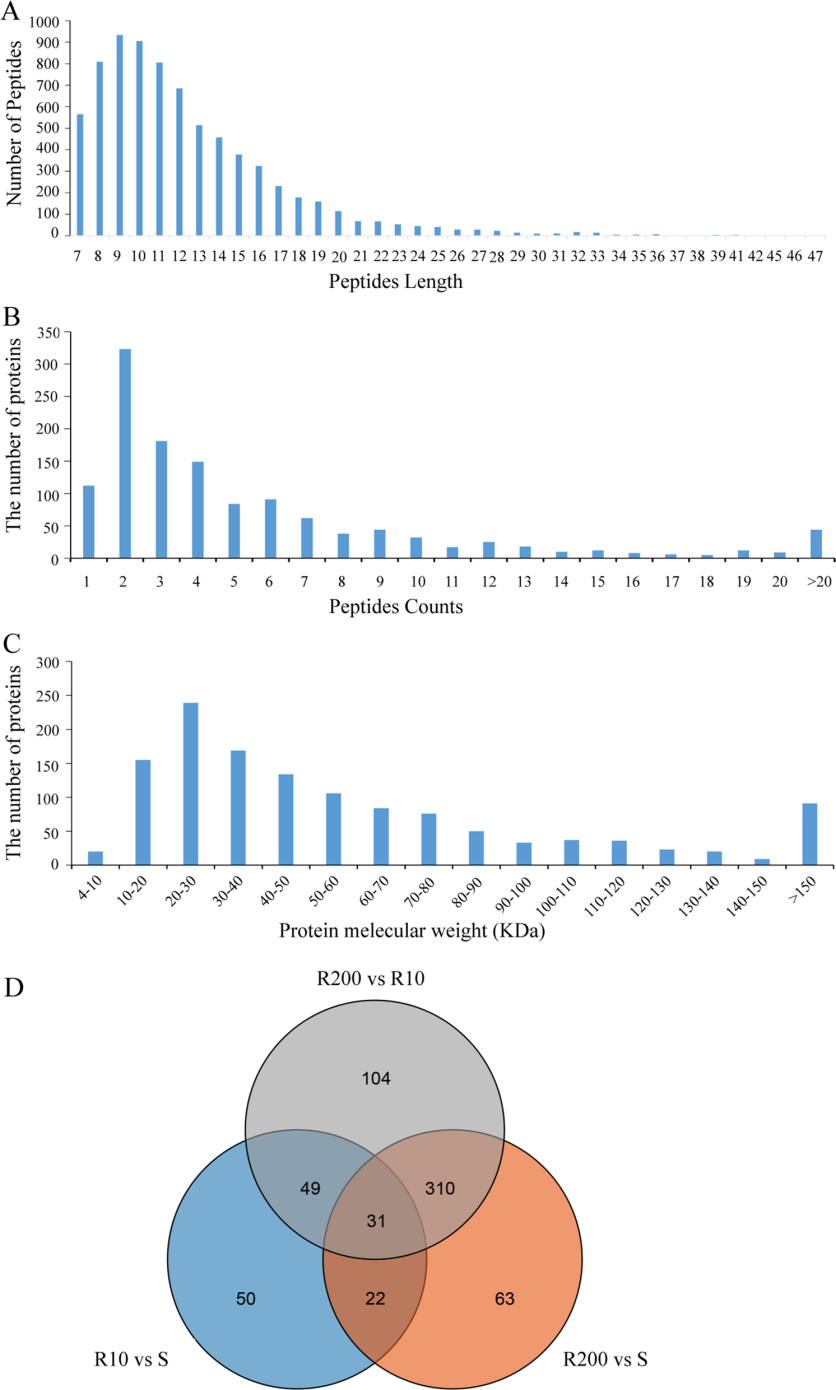
Summary of the label-free proteomic data. **A** Distribution of identified peptides in terms of their length, **B** Distribution of proteins according to number of peptides, **C** Distribution of proteins based on their molecular weight, **D** Venn diagram of the differentially expressed proteins among groups S, R10 and R200. Abbreviations: S, Control strain, ethanamizuril (EZL) sensitive; R10, EZL-resistant strain induced by a constant 10 mg EZL/kg poultry feed; R200: EZL-resistant strain induced by gradually increasing dosages of EZL

A total of 7511 peptides (Additional file 2: Table S6) and 1282 proteins (Additional file 3: Table S7) were identified using the label-free proteomic approach. The distribution of peptide lengths is shown in Fig. 1A, and the distribution of the number of proteins based on the number of peptides that matched with them is shown in Fig. 1B. The distribution of the identified proteins in terms of their molecular weight is shown in Fig. 1C. Figure 1D shows the DEPs found between the different groups. We identified 152, 426 and 494 DEPs in the R10 versus S groups, R200 versus S groups and R200 versus R10 groups, respectively (Fig. 1D; Additional file 1: Table S8).

### Bioinformatics analyses of DEPs

**Fig. 2:**
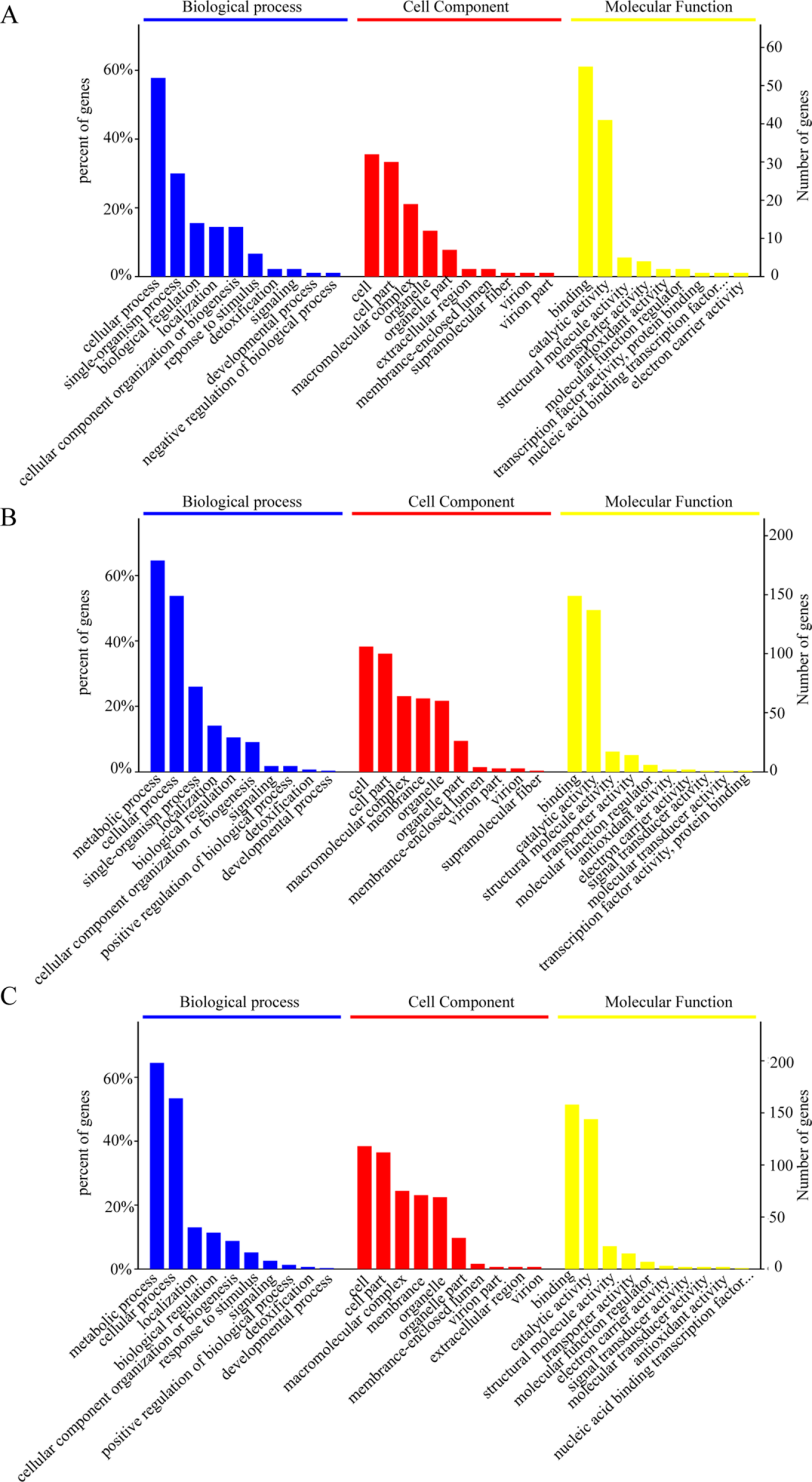
Gene ontology annotation of differentially expressed proteins in the comparison groups. **A** Comparison of R10 vs S groups, **B** Comparison of R200 vs S groups, **C ** Comparison of R200 vs R10 groups

To better understand the roles of the DEPs in the resistance mechanisms of *E. tenella*, GO and KEGG pathway analyses were performed on the DEPs of three comparison groups: R10 versus S, R200 versus S and R200 versus R10. The GO results are shown in Fig. 2. GO predictions were identified for 127 (83.5%), 356 (83.5%) and 400 (80.9%) of the R10 versus S, R200 versus S and R200 versus R10 comparison groups, respectively. The enriched GO terms of DEPs in the three paired comparisons were similar. The DEPs of biological processes were mainly associated with metabolic and cellular processes. The top two common cell component categories were cell and cell parts. The top two common molecular function categories were mainly involved in binding and catalytic activity.

**Fig. 3:**
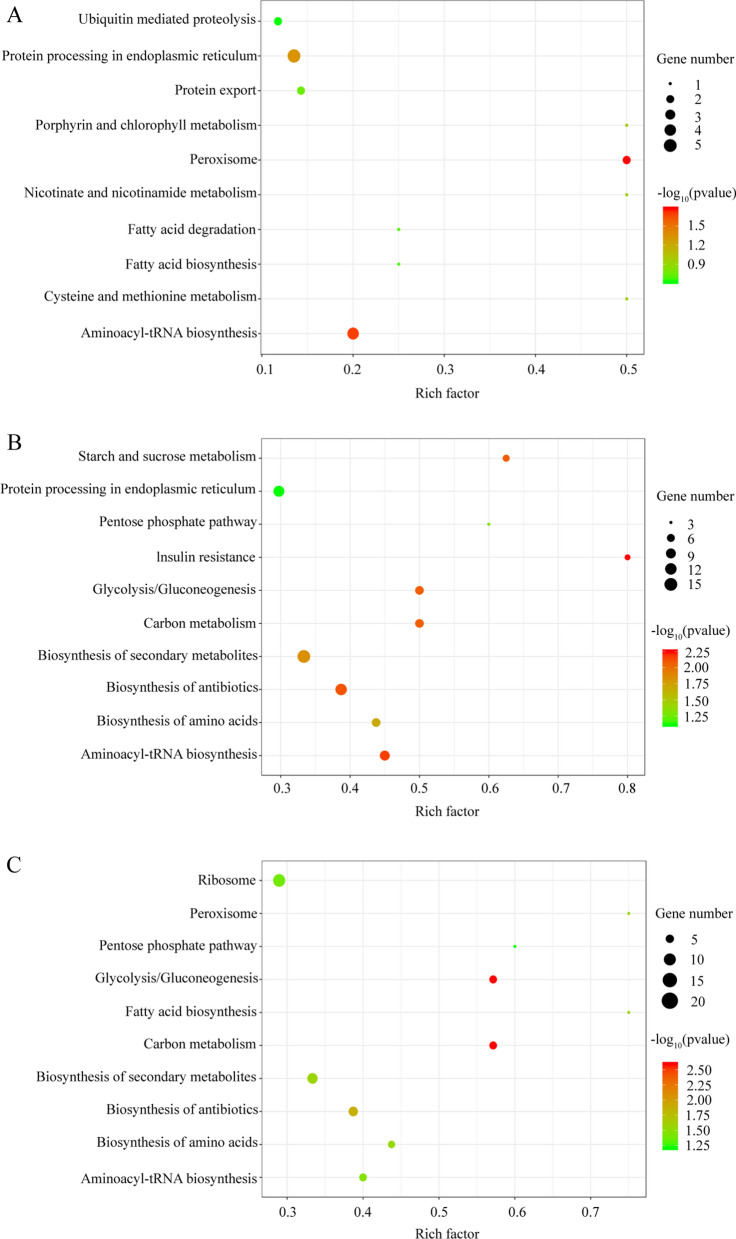
Kyoto Encyclopedia of Genes and Genomes pathway enrichment analysis of differentially expression proteins in the comparison groups. **A** Comparison of R10 vs S, **B ** Comparison of R200 vs S, **C** Comparison of R200 vs R10

KEGG analysis revealed 34, 118 and 126 proteins that were annotated and enriched in 28, 52 and 54 KEGG pathways, respectively, in the comparison groups of R10 versus S, R200 versus S and R200 versus R10, respectively. In the R10 versus S comparison group, the common dominant KEGG pathways were peroxisome and aminoacyl-transfer RNA (tRNA) biosynthesis and protein processing in the endoplasmic reticulum. DEPs in the R200 versus S comparison group were associated with insulin resistance; aminoacyl-tRNA and antibiotic biosynthesis; starch, sucrose and carbon metabolism; and glycolysis/gluconeogenesis pathways. The top two enriched pathways were carbon metabolism and glycolysis/gluconeogenesis pathways in the R200 versus R10 comparison group. KEGG pathway analysis results of DEPs are shown in Fig. 3.

### Common differential protein analyses

**Fig. 4:**
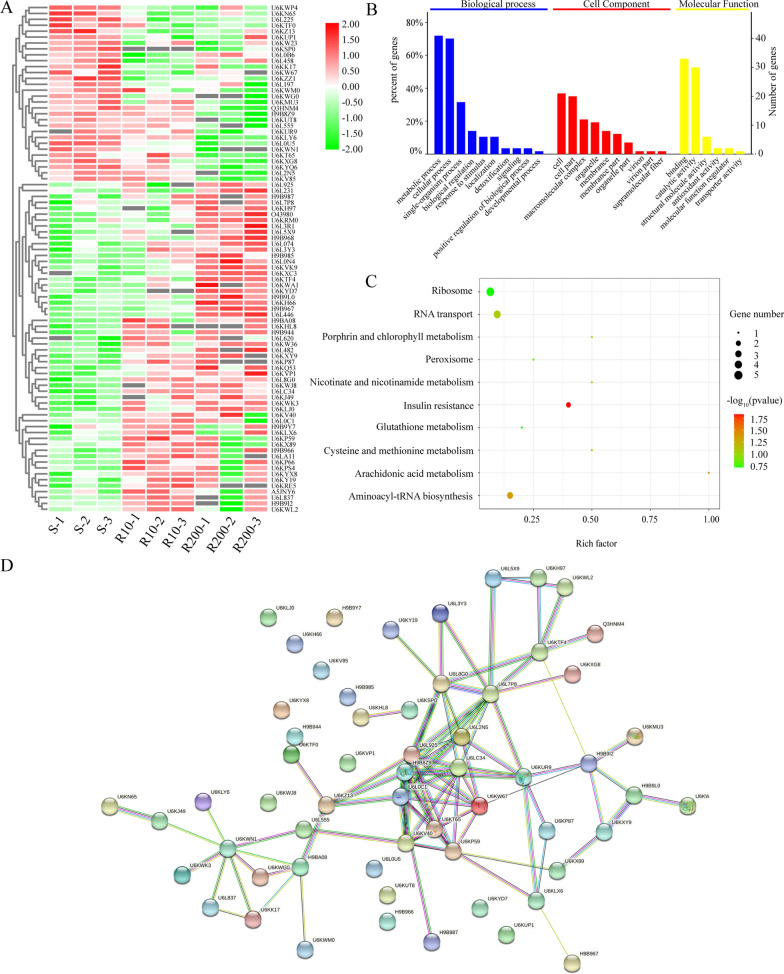
Bioinformatics analyses of the 86 common differentially expression proteins in the two EZL-resistant strains (R10, R200) compared with the EZL-sensitive strain (S). **A** Hierarchical clustering analysis, **B** Gene Ontology analysis, **C** Kyoto Encyclopedia of Genes and Genomes pathway enrichment analysis, **D** Protein–protein interaction network analysis

We selected 86 proteins that were detected in the comparison of R10 versus S and R200 versus S groups, with the aim to perform further analysis due to the similar trends in their expression patterns and significant differences in at least one of these comparisons. Within this set, 56 proteins were upregulated, and 30 proteins were downregulated (Fig. 4A). These 86 proteins were annotated based on biological processes, cell components and molecular functions (Fig. 4B). In the biological process class, proteins were mainly associated with metabolic and cellular processes. The proteins of the cellular component class were mainly involved in the cell and cell parts. In terms of molecular function, most proteins were distributed in the binding and catalytic activity categories. KEGG pathway analysis results of these 86 proteins are shown in Fig. 4C. These proteins are mainly linked to insulin resistance, arachidonic acid metabolism and aminoacyl-tRNA biosynthesis pathways. Protein–protein interaction (PPI) network analysis of the common DEPs was performed using the STRING database. Fifty-nine DEPs were identified in the 86 DEP-matched PPI networks (Fig. 4D), of which 10 were found to be hubs in the functional interaction network (≥ 8 edges): (Uniprot: U6L0C1, 40S ribosomal protein S20, putative; H9B8Z9, 40S ribosomal protein SA; U6L925, 60S ribosomal protein L13a; U6L7P8, TCP-1/cpn60 family chaperonin, putative; U6LC34, 60S ribosomal protein L30, putative; U6KW67, 40S ribosomal protein S17; U6KV40, glutamyl-tRNA synthetase, putative; U6L8G0, T-complex protein 1 subunit delta; U6KP59, nascent polypeptide-associated complex subunit beta; and U6KWN1, equisetin synthetase). Additionally, six proteins (Uniprot: U6L7P8, U6L5X9, H9B8Z9, U6L8G0, H9B987 and U6KWP4) were associated with the cytoplasm, and four proteins (Uniprot: U6KK17, U6L837, H9BA08, and U6KZ13) were found to be enriched in peroxisomes.

### Validation of messenger RNA abundance

**Fig. 5:**
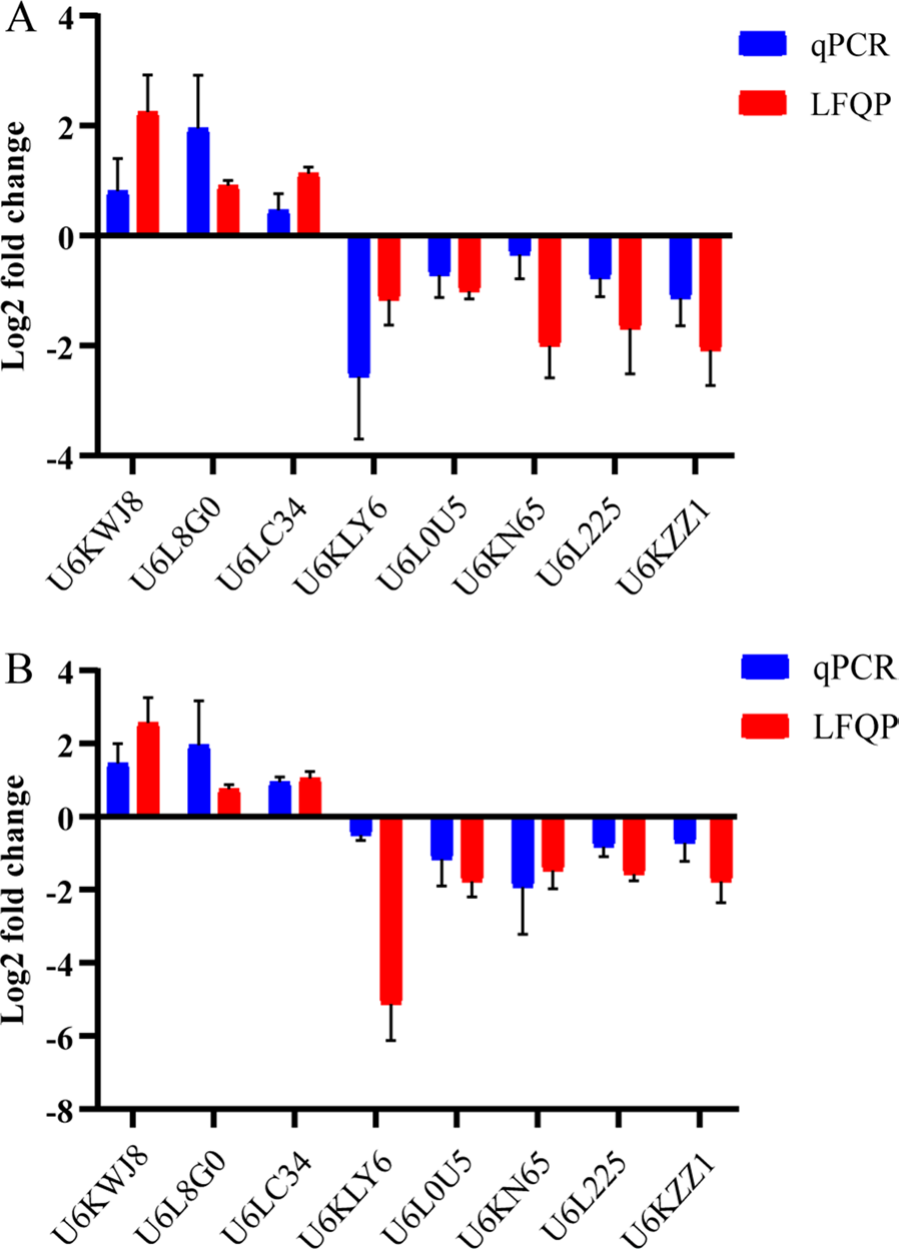
Assessment of gene expression by qPCR of eight genes selected from the label-free proteomic results. **A** R10 vs S comparison group, **B** R200 vs S comparison group. Abbreviations: LFQP, Label-free quantitative proteomic (analysis); qPCR, quantitative real-time PCR

We chose eight common DEPs in the R10 versus S and R200 versus S comparison groups (Uniprot: U6KWJ8, U6L8G0, U6LC34, U6KLY6, U6L0U5, U6KN65, U6L225, U6KZZ1) to examine the levels of steady-state messenger RNA (mRNA) in the three strains. The mRNA levels showed trends that were consistent with the label-free proteomic data (Fig. 5).

## Discussion

In this study, we induced two resistant *E. tenella* strains against different EZL dosages and studied oocyst production and sporulation of the sensitive and resistant strains. We also compared the proteomic profiles of these strains to identify the proteins responsible for EZL resistance. Our aim was to provide a new perspective on and understanding of proteins related to the mechanisms of drug resistance of *E. tenella* against anticoccidial drugs.

According to the results of the drug resistance evaluation test, strain R10 was completely resistant to 10 mg EZL/kg (poultry feed), and strain R200 was completely resistant to 200 mg EZL/kg. The total oocyst output decreased with increased drug resistance. However, increased drug resistance had minimal influence on the sporulation percentage. Phenotypic studies of resistant strains may help to explain the mechanism of drug resistance development in *Eimeria* spp.

The 86 common DEPs identified in this study were enriched in aminoacyl-tRNA biosynthesis, RNA transport, ribosomes and metabolic pathways. Aminoacyl-tRNA synthetases are vital components of the translational machinery as they catalyze amino acids to specific cognate tRNAs. Recently, aminoacyl-tRNA synthetases have been explored as anti-parasitic drug targets for diseases such as malaria, toxoplasmosis, leishmaniasis, cryptosporidiosis and coccidiosis, as they can effectively inhibit the growth of parasites [18–20]. Additionally, it has been shown that amino acid mutations in the gene encoding methionyl-tRNA synthetase result in drug resistance in *Cryptosporidium parvum* [21]. Therefore, it is possible that proteins in the aminoacyl-tRNA biosynthesis pathway are related to the development of drug resistance of *E. tenella* against EZL. The PPI functional network of the 86 common DEPs showed 10 important hub proteins, of which most were ribosomal proteins associated with the ribosome pathway. In summary, the transcriptional and translational processes may be involved in the development of drug resistance of *E. tenella* against EZL.

We identified DEPs involved in ATP and GTP binding, invasion and membrane components (Additional file 1: Table S9). We found that most of the ATP and GTP binding proteins were upregulated, suggesting that maintaining cellular energy may be one component of resistance. There were significant differences in the T complex protein-1 (TCP-1) in strains the R10 and R200 strains (upregulated) compared with the S strain. This result is similar to that of amphotericin B resistance in *Leishmania infantum* [22]. TCP-1 has functions in ATP processes and unfolded protein binding, and may also play critical roles in regulating the cell cycle and cytoskeleton. When *Musca domestica* larvae were challenged with microbes or received a short-term heat shock, *Md*TCP-1f mRNA levels increased, suggesting that *Md*TCP-1f performs an immune defense function [23]. TCP-1 also functions as a molecular chaperon with chaperonin-containing tail-less complex polypeptide 1 (CCT), which is a necessary polypeptide in spermatogenesis in planarian flatworms and is a highly conserved hetero-oligomer that ensures proper folding of actin, tubulin and mitotic regulators [24]. Furthermore, in breast cancer cells, TCP-1 is an intracellular target of CT20p, as demonstrated in a study where alteration in the levels of the CCT ß-subunit resulted in altered susceptibility to CT20p [25]. The roles of TCP-1 in cell division, cycle control, cytoskeleton and immune defense may combine to affect resistance in *E. tenella*.

We also identified proteins with Ras family domains possessing GTPase activity that were significantly upregulated in the resistant strains (R) compared with their counterparts in in the sensitive strain (S). Upregulated Ras family members in *Toxoplasma gondii* were reported to contribute to resistance against monensin [6]. Ras-related proteins are also involved in cell proliferation, apoptosis inhibition and resistance to vemurafenib (PLX4032) via MAPK pathway reactivation [26, 27]. Therefore, Ras-related proteins may play roles in the proliferation and differentiation of coccidium and in the development of drug resistance of *E. tenella* against EZL.

Invasion of apicomplexan parasites begins with the secretion of several groups of microneme proteins that form host–pathogen adhesion complexes consisting of microneme proteins and surface antigens. The parasite then moves across the host cell membrane and forms a parasitophorous vacuole (PV) in the host cell. Rhoptry proteins (ROPs) are delivered to the periplasmic surface of the PV and the host cell nucleus to accomplish host immune evasion and intracellular survival. These proteins also regulate host cell signaling pathways and downstream gene expression [28–30]. The microneme and ROP levels were reduced or dissipated after EZL treatment in second-generation schizonts and merozoites, indicating that EZL most likely disturbs the invasion process of *E. tenella* [31]. In the present study, microneme protein 2 (MIC2) was upregulated in strain R10 and significantly upregulated in strain R200 compared with strain S. MIC2 is a 50-kDa acidic protein in the microneme organelles of sporozoites and merozoites and is crucial for the interaction between the parasite and host at the time of invasion [32]. MIC4 was reported to be downregulated in monensin-resistant *E. tenella* strains, which may indicate a lower invasion activity with resistance [5]. Moreover, in response to monensin, MIC8 is downregulated in the *T. gondii* strain RH [6]. MIC2 was also downregulated in a sulfadiazine-resistant *T. gondii* strain ME49 and in an *E. tenella* strain resistant to maduramicin [33, 34]. Furthermore, MIC proteins are generally downregulated in *E. tenella*, which is resistant to polyether ionophores. Conversely, in the present study MIC2 was upregulated in resistant strains R10 and R200, possibly in association with the increased invasion activity in response to EZL. ROP25 is a rhoptry kinase family member that was upregulated in both resistant strains. An increase in ROP18 corresponded to a higher resistance of *T. gondii* strain RH to monensin [6]. In addition, ROP25 upregulation in our resistant strains may lead to increased invasion by *E. tenella*. Surface proteins of apicomplexan parasites are linked to glycosylphosphatidyl inositol; these surface proteins include the surface antigen (SAG) proteins that are involved in host cell adhesion and invasion [4, 35, 36]. We found that SAG family members were upregulated in the EZL-resistant strains. A similar finding was observed in *T. gondii* strain RH in response to monensin [6]. However, *SAG13* and *SAG10* were downregulated in diclazuril- and maduramicin-resistant *E. tenella* at the transcription level [34]. In the present study, the increase in SAG family members might enhance the invasion activity of coccidium sporozoites.

Adenosylhomocysteinase was downregulated in the resistant strains, whereas it was upregulated in *Leishmania infantum* showing resistance to amphotericin B[22]. This enzyme catalyzes the reversible hydrolysis of* S*-adenosyl-L-homocysteinase (SAH) to adenosine and L-homocysteine, and its inhibition results in SAH accumulation. This gene is frequently amplified in human malignant cancers, is upregulated in tumors and is a validated anti-tumor target [37]. Some nucleoside analogs that inhibit enzyme activity are parasite growth inhibitors [38]. The downregulation of adenosylhomocysteinase may influence coccidia growth in the development of *E. tenella* resistance to EZL.

Most of the membrane proteins found in strains R10 and R200 were upregulated and are integral membrane components, which may be related to transporter functions. These are additional targets for research into the drug resistance mechanisms of *E. tenella* against EZL.

Although some confirmed drug-target proteins in* Plasmodium* are upregulated at the protein or transcription levels, some remain unchanged (Additional file 1: Table S10) [39–44]. A study on the chloroquine resistance transporter (*pf*crt) gene in *Plasmodium falciparum* showed that there has no relationship between the expression level and response to the drug [44]. Interestingly, all of drug target genes were mutated (Additional file 1: Table S10) and some of them were accompanied by increases in copy number. Therefore, we speculated that the resistance mechanism for R10 and R200 was caused by more mutations in the gene of the target protein. However, variation in copy numbers cannot be ruled out. Nevertheless, there are a few limitations in the design of this research. Although each strain had three biological replicates and one technical duplication, there should have been random up- or downregulated proteins because of random mutation.

### Conclusion

In conclusion, we found that with increased resistance, total oocyst output decreases but sporulation is unaffected. We also detected 86 proteins with common expressional trends in the resistant strains, most of which play vital roles in pathogenicity and biological functioning. These results indicate that the resistance mechanisms of *E. tenella* against EZL might be related to the transcriptional and translational processes, especially in the factors that inhibit the growth of parasites. The significant number of DEPs found in this study provide new insights into the resistance mechanisms of *E. tenella* against EZL, and provide a basis for further evaluation of new chemotherapeutic targets to control *E. tenella* infections.

## Supplementary Information

**Additional file 1: **Figure S1. The PCR identification results of single oocyst strains. Figure S2. Oocyst production of the ethanamizuril sensitive (S) and resistant strains (R10 and R200). Figure S3. Oocyst sporulation of the ethanamizuril sensitive (S) and resistant strains (R10 and R200). Table S1. The schedule for increasing the EZL dosage from 3 to 200 mg/kg in the chicken feed. Table S2. The categorization of 10 groups of drug resistance evaluation test. Table S3. Primers used for qRT-PCR validation. Table S4. The lesion value, oocyst value and ACI value of Eimeria tenella strains against ethanamizuril. Table S5. The percentage of sporulated oocysts of the ethanamizuril sensitive (S) and resistant strains (R10 and R200). Table S8. Differentially expressed proteins in each comparison group. Table S9. Statistical analysis of 86 differentially expressed proteins in R10 vs. S and R200 vs. S comparison groups. Table S10. The reported drug-target in apicomplexan parasites.  Supplementary material S1：The calculated method of the four anticoccodial indices.

**Additional file 2: **Table S6.  Peptides identified by label-free quantitative proteomic analysis of the ethanamizuril sensitive and resistant Eimeria tenella strains.

**Additional file 3: ** Table S7. Proteins identified by label-free quantitative proteomic analysis of ethanamizuril sensitive and resistant Eimeria tenella strains.

## Data Availability

All data used in this study are freely accessible, and can be found in the manuscript and in Additional files 1 ,2 and 3 2.

## Abbreviations

ACI: Anticoccidial index
DEPs: Differentially expressed proteins
EZL: Ethanamizuril
GO: Gene Ontology
KEGG: Kyoto Encyclopedia of Genes and Genomes (KEGG)
LFQ: Label-free quantification
POAA: Percentage of optimum anticoccidial activity
RLS: Reduction in lesion scores
ROP: Relative oocyst production
PPI: Protein-protein interaction
SD: Standard deviation
mRNA: Messenger RNA
rRNA: Ribosomal RNA
SAG: Surface antigen
ROPs: Rhoptry proteins
qPCR: quantitative real-time PCR
