# Supplementary material for: Label-free quantitative proteomic analysis of ethanamizuril-resistant versus -sensitive strains of Eimeria tenella
Source: Parasit Vectors. 2022 Sep 8;15:319. doi: 10.1186/s13071-022-05412-6 (PMC9454127; doi:10.1186/s13071-022-05412-6)
Supplement: Supplementary file 1 — Additional file 1: Figure S1. The PCR identification results of single oocyst strains. Figure S2. Oocyst production of the ethanamizuril sensitive (S) and resistant strains (R10 and R200). Figure S3. Oocyst sporulation of the ethanamizuril sensitive (S) and resistant strains (R10 and R200). Table S1. The schedule for increasing the EZL dosage from 3 to 200 mg/kg in the chicken feed. Table S2. The categorization of 10 groups of drug resistance evaluation test. Table S3. Primers used for qRT-PCR validation. Table S4. The lesion value, oocyst value and ACI value of Eimeria tenella strains against ethanamizuril. Table S5. The percentage of sporulated oocysts of the ethanamizuril sensitive (S) and resistant strains (R10 and R200). Table S8. Differentially expressed proteins in each comparison group. Table S9. Statistical analysis of 86 differentially expressed proteins in R10 vs. S and R200 vs. S comparison groups. Table S10. The reported drug-target in apicomplexan parasites. Supplementary material S1：The calculated method of the four anticoccodial indices. [file 13071_2022_5412_MOESM1_ESM.doc]

Supplementary Material

**Supplementary Figures and Tables**

Figure S1.

Figure S2.

Figure S3.

Table S1.

Table S2.

Table S3.

Table S4.

Table S5.

Table S6.

Table S7.

Table S8.

Table S9.

Table S10.

Supplementary material S1


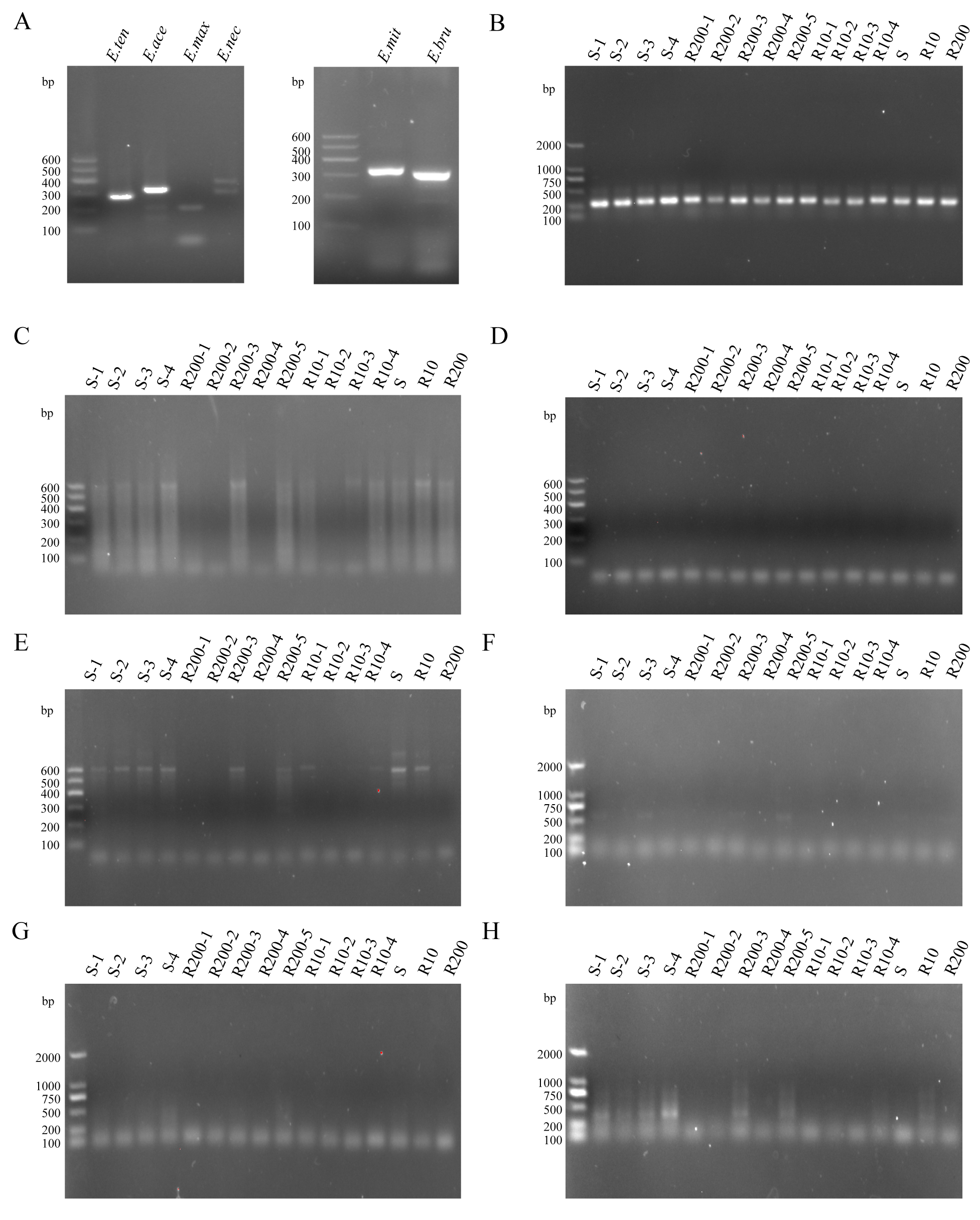


**Figure S1**. The PCR identification results of single oocyst strains. (A) Positive control. *E.ten*: *Eimeria tenella*; *E.ace*: *Eimeria acervulina*; *E.max*: *Eimeria maxima*; *E.nec*: *Eimeria necatrix*; *E.mit*: *Eimeria mitis*; *E.bru*: *Eimeria brunetti*. We have not the genome of *Eimeria praecox* strain, so we did not amplify positive control for it. (B) The PCR amplification results using the primers of *E.tenella*; (C) The PCR amplification results using the primers of *E.acervulina*; (D) The PCR amplification results using the primers of *E.maxima*; (E) The PCR amplification results using the primers of *E.necatrix*; (F) The PCR amplification results using the primers of *E.brunetti*; (G) The PCR amplification results using the primers of *E.mitis*; (H) The PCR amplification results using the primers of *E.praecox*. Single oocyst strains of sensitive stains: S, S-1, S-2, S-3, S-4; Single oocyst strains of R10 stains: R10, R10-1, R10-2, R10-3, R10-4; Single oocyst strains of R200 stains: R200, R200-1, R200-2, R200-3, R200-4, R200-5. At last, we selected the single oocyst strains of S, R10 and R200 for further study.


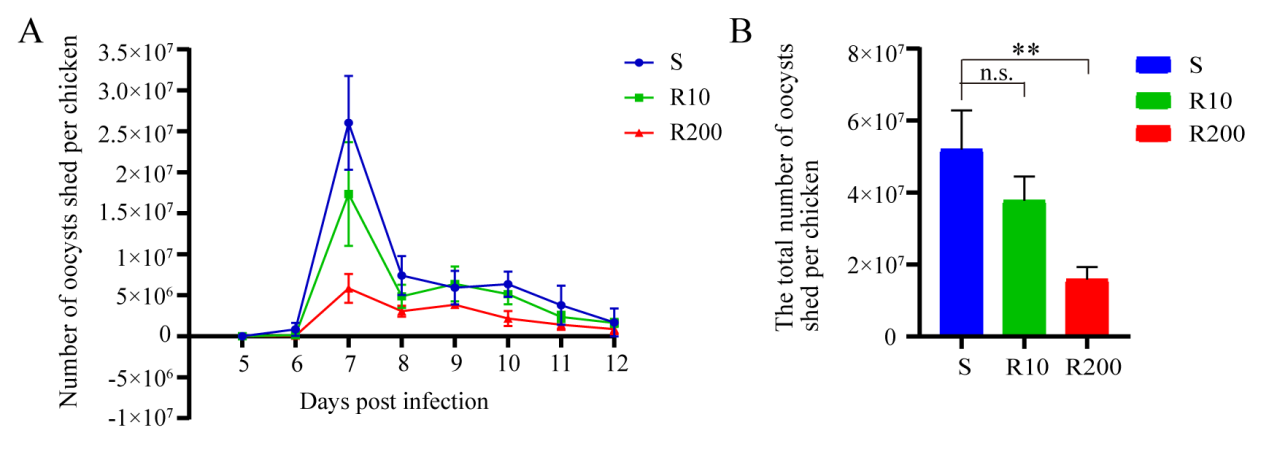


**Figure S2**. Oocyst production of the ethanamizuril sensitive (S) and resistant strains (R10 and R200). (A) The oocyst output curves of each strain. (B) The total number of oocysts shed per chicken from each strain. The results were presented as mean ± SD in triplicates.


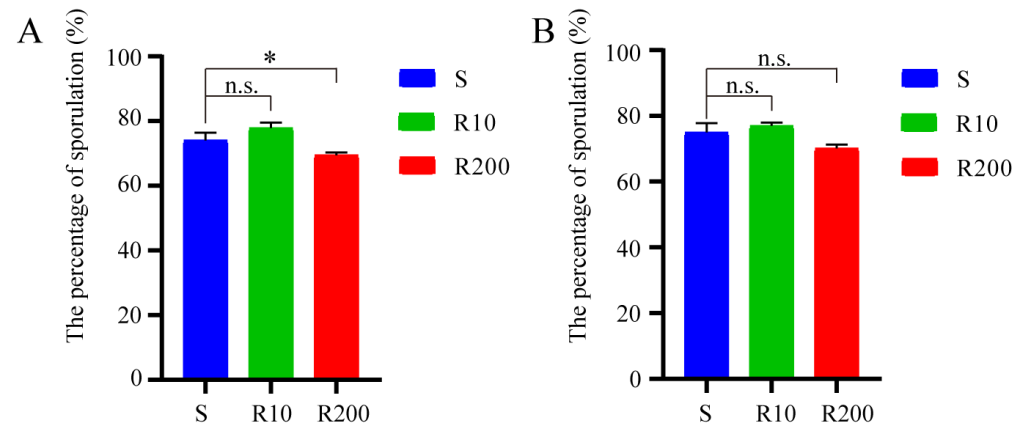


**Figure S3**. Oocyst sporulation of the ethanamizuril sensitive (S) and resistant strains (R10 and R200). The percentage of sporulation of the strains after (A) 24 h and (B) 48 h of sporulation.

**Table S1**. The schedule for increasing the EZL dosage from 3 to 200 mg/kg in the chicken feed.

| EZL Dosage (mg/kg) | 3 | 5 | 10 | 20 | 40 | 60 | 80 | 100 | 120 | 200 |
| --- | --- | --- | --- | --- | --- | --- | --- | --- | --- | --- |
| Number of Passages | 2 | 1 | 7 | 2 | 2 | 1 | 2 | 2 | 2 | 6 |

EZL: ethanamizuril.

**Table S2**. The categorization of 10 groups of drug resistance evaluation test

| Groups | 1 | 2 | 3 | 4 | 5 | 6 | 7 | 8 | 9 | 10 |
| --- | --- | --- | --- | --- | --- | --- | --- | --- | --- | --- |
| Infect strain | none | S | R10 | R200 | S | S | R10 | R10 | R200 | R200 |
| EZL dosage (mg/kg) | none | none | none | none | 10 | 200 | 10 | 200 | 10 | 200 |

S: EZL sensitive strain; R10: EZL resistant strain induced by a constant 10 mg/kg dose of EZL. R200: EZL resistant strain induced by gradually increasing dosages of EZL; EZL: ethanamizuril.

**Table S3**. Primers used for qRT-PCR validation.

| Proteins | Gene ID | Primer name | Primer sequence (5' to 3') | Product length (bp) |
| --- | --- | --- | --- | --- |
| U6KWJ8 | 25249256 | U6KWJ8-F | TTCTCTCACTTGCTGCTCCG | 225 |
|  |  | U6KWJ8-R | TCGTGGCCAAGACCATCTTC |  |
| U6L8G0 | 25249323 | U6L8G0-F | TACACTTTGGCTGAGAACG | 168 |
|  |  | U6L8G0-R | CTGGGAACCTGACACTCG |  |
| U6LC34 | 25249267 | U6LC34-F | GCGAAGAAGCAGAAGCAAGG | 194 |
|  |  | U6LC34-R | TTGGCCAGCATTGCGTAGTA |  |
| U6KLY6 | 25254943 | U6KLY6-F | TTGGGCTTATTGGACGTCCC | 150 |
|  |  | U6KLY6-R | TCTGGCTGGATGCAGTCAAG |  |
| U6L0U5 | 25250379 | U6L0U5-F | TTTTCCGCCGAACACTTT | 163 |
|  |  | U6L0U5-R | CTCGCCTCGAATGCTCTT |  |
| U6KN65 | 25254515 | U6KN65-F | TCATTGAAACCCTCACCCAC | 238 |
|  |  | U6KN65-R | TGAATCAGCATTGTTATGTCCC |  |
| U6L225 | 25254781 | U6L225-F | GGTGTGGTAGGCCTCTTTCC | 183 |
|  |  | U6L225-R | GCTGCACTGACCTGTGAGAT |  |
| U6KZZ1 | 25249252 | U6KZZ1-F | CGCAACTCAGGTGGTAGAGT | 225 |
|  |  | U6KZZ1-R | GGCCCCTGTTCTATGAGGTG |  |
| 18S | KT184354.1 | 18S-F | ATCGCAGTTGGTTCTTTTGG | 170 |
|  |  | 18S-R | CCTGCTGCCTTCCTTAGATG |  |

**Table S4**. The lesion value, oocyst value and ACI value of *Eimeria tenella* strains against ethanamizuril.

| Strains | Dose (mg/kg) | Lesion value (Mean ± SD) | Oocyst value (Mean ± SD) | ACI (Mean ± SD) |
| --- | --- | --- | --- | --- |
| S | 0 | 20 ± 1.77 | 30 ± 14.14 | 92.43 ± 34.95 |
|  | 10 | 7.85 ± 1.39 | 1.67 ± 2.88 | 199.43 ± 11.16 |
|  | 200 | 0.00 ± 0.00 | 0.00 ± 0.00 | 195.74 ± 4.14 |
| R 10 | 0 | 18.75 ± 3.53 | 30 ± 14.14 | 108.66 ± 6.07 |
|  | 10 | 17.08 ± 2.88 | 26.67 ± 11.54 | 118.53 ± 14.55 |
|  | 200 | 2.5 ± 1.25 | 3.33 ± 2.88 | 180.3 ± 4.37 |
| R 200 | 0 | 20 ± 0.00 | 40 ± 0.00 | 110.82 ± 6.85 |
|  | 10 | 15.42 ± 0.72 | 40 ± 0.00 | 122.49 ± 4.90 |
|  | 200 | 17.5 ± 1.25 | 40 ± 0.00 | 89.14 ± 13.00 |

**Table S5**. The percentage of sporulated oocysts of the ethanamizuril sensitive (S) and resistant strains (R10 and R200).

| Groups | 24 h (%) | 48 h (%) |
| --- | --- | --- |
| S | 74.00±2.49 | 74.96±2.83 |
| R10 | 77.87±1.68 | 76.97±0.97 |
| R200 | 69.47±0.79 | 70.11±1.14 |

**Table S6**. Peptides identified by label-free quantitative proteomic analysis of the ethanamizuril sensitive and resistant *Eimeria tenella* strains.

Excel Table S6.

**Table S7**. Proteins identified by label-free quantitative proteomic analysis of ethanamizuril sensitive and resistant *Eimeria tenella* strains.

Excel Table S7.

**Table S8**. Differentially expressed proteins in each comparison group.

| Compare | Differentially expressed proteins (up/down) | §Presence in each group | Total differential proteins |
| --- | --- | --- | --- |
| R10 vs S | 95(68/27) | 57(38/19) | 152 |
| R200 vs S | 108(67/41) | 318(18/300) | 426 |
| R200 vs R10 | 124(64/60) | 370(19/351) | 494 |

§, Numbers in parentheses indicate the number of proteins in listed first and second in the compare column, respectively on each side of the slash.

‘S’denotes an EZL sensitive *E.tenella* strain and ‘R10’ and ‘R200’ represent EZL resistant *E.tenella* strains passaged in chickens (see text for details). The differentially proteins were assessed according to P value < 0.05 and ratio > 1.5 or < 0.667.

**Table S9**. Statistical analysis of 86 differentially expressed proteins in R10 vs. S and R200 vs. S comparison groups.

| Protein ID | Description | Average R 10 /S | t-test P value (R10 vs S) | Average R 200 /S | t-test P value (R200 vs S) | Biological process | Cellular component | Molecular function |
| --- | --- | --- | --- | --- | --- | --- | --- | --- |
| U6KWJ8 | Uncharacterized protein | 5.0257 | 0.0443 | 6.3456 | 0.0252 | - | membrane | - |
| U6KXY9 | Ras family domain-containing protein, putative | 2.6796 | 0.0132 | 4.6454 | 0.0026 | - | - | GTPase activity, GTP binding. |
| U6L482 | Uncharacterized protein | 1.7688 | 0.0227 | 3.6628 | 0.0080 | - | - | - |
| U6L8G0 | T-complex protein 1 subunit delta | 1.8811 | 0.0053 | 1.6864 | 0.0139 | [protein folding](https://www.ebi.ac.uk/QuickGO/term/GO:0006457) | cytoplasm | [ATP binding](https://www.ebi.ac.uk/QuickGO/term/GO:0005524) [unfolded protein binding](https://www.ebi.ac.uk/QuickGO/term/GO:0051082) |
| U6LC34 | 60S ribosomal protein L30, putative | 2.1944 | 0.0026 | 2.0724 | 0.0071 | - | ribosome | - |
| O43980 | Mannitol-1-phosphatase | 1.1685 | 0.3029 | 2.0595 | 0.0052 | mannitol biosynthetic process | - | manntitol-1-phosphatase activity. |
| H9B944 | Uncharacterized protein | 1.7966 | 0.1747 | 2.5396 | 0.0014 | - | - | - |
| H9B967 | p25-alpha domain-  containing protein, putative | 1.4365 | 0.2943 | 8.2619 | 0.0036 | - | - | - |
| H9B968 | Uncharacterized protein | 1.2197 | 0.4800 | 2.6533 | 0.0203 | - | - | - |
| H9B985 | Uncharacterized protein | 1.4391 | 0.1570 | 2.1422 | 0.0337 | - | - | - |
| H9B987 | Methionine aminopeptidase 2 | 1.8148 | 0.1824 | 2.5442 | 0.0310 | [protein initiator methionine removal](https://www.ebi.ac.uk/QuickGO/term/GO:0070084) | Cytoplasm | [metal ion binding](https://www.ebi.ac.uk/QuickGO/term/GO:0046872)[metalloaminopeptidase activity](https://www.ebi.ac.uk/QuickGO/term/GO:0070006) |
| H9B9L0 | Uncharacterized protein | 1.3412 | 0.3745 | 3.1640 | 0.0339 | - | - | GTPase activity, GTP binding |
| H9BA08 | Uncharacterized protein | 2.6086 | 0.1560 | 2.4503 | 0.0118 | isocitrate metabolic process |  | isocitrate dehydrogenase(NADP+) activity,  magnesium ion binding, NAD binding |
| U6KH66 | Rhoptry kinase family protein ROP25, putative | 1.0922 | 0.6648 | 3.4061 | 0.0084 | - | - | ATP binding. Protein kinase activity |
| U6KJ49 | O-acetylserine (Thiol) lyase, putative | 1.8480 | 0.1150 | 1.9902 | 0.0265 | - | - | lyase activity. |
| U6KLJ0 | Uncharacterized protein | 2.2613 | 0.1047 | 3.4451 | 0.0009 |  | membrane |  |
| U6KRM0 | SAG family member | 1.0784 | 0.8345 | 2.2999 | 0.0133 | - | - | - |
| U6KTF4 | Serine/threonine-protein phosphatase | 2.1005 | 0.2055 | 6.0577 | 0.0001 | - | - | calcium ion binding, Phosphoprotein phosphatase activity |
| U6KVK9 | SAG family member | 1.2149 | 0.4319 | 2.9810 | 0.0026 | - | - | - |
| U6KVP1 | Membrane-attack complex / perforin domain-containing protein, putative | 1.4094 | 0.0922 | 1.9364 | 0.0465 | - | - | ATP binding, protein kinase activity |
| U6KW36 | Uncharacterized protein | 1.7635 | 0.0676 | 1.9878 | 0.0102 |  | membrane |  |
| U6KWA1 | Uncharacterized protein | 1.2934 | 0.1965 | 2.3011 | 0.0308 | - | - | - |
| U6KWK3 | Succinate-semialdehyde dehydrogenase, putative | 1.7082 | 0.0642 | 2.0077 | 0.0151 | - | - | oxidoreductase activity |
| U6KYD7 | Uncharacterized protein | 1.0265 | - | 8.6985 | 0.0052 | - | - | - |
| U6L074 | Uncharacterized protein | 1.5115 | 0.1721 | 2.1169 | 0.0336 | - | - | - |
| U6L0N4 | Uncharacterized protein | 1.3997 | 0.1497 | 2.4990 | 0.0206 | - | - | - |
| U6L231 | Uncharacterized protein | 1.1113 | 0.8663 | 3.9314 | 0.0475 | - | - | - |
| U6L3R1 | SAG family member | 1.1321 | 0.4896 | 2.1442 | 0.0335 | - | membrane | - |
| U6L3Y3 | Prohibitin, putative | 1.5825 | 0.2027 | 1.8193 | 0.0318 | - | membrane | - |
| U6L446 | Uncharacterized protein | 1.0615 | 0.3531 | 1.9933 | 0.0007 | - | - | - |
| U6L5X9 | Proteasome subunit beta type, related | 1.8101 | 0.1527 | 3.0065 | 0.0351 | [proteolysis involved in cellular protein catabolic process](https://www.ebi.ac.uk/QuickGO/term/GO:0051603) | nucleus, proteasome core complex | [threonine-type endopeptidase activity](https://www.ebi.ac.uk/QuickGO/term/GO:0004298) |
| U6L7P8 | TCP-1/cpn60 family chaperonin, putative | 1.4119 | 0.4430 | 2.4764 | 0.0466 | protein folding | - | ATP binding，unfolding protein binding. |
| H9B966 | Chromosome II, complete genome, related | 3.1343 | 0.0037 | 2.6291 | 0.1975 | - | membrane | - |
| U6KLX6 | Glycine-rich protein 2, putative | 1.9438 | 0.0167 | 1.3068 | 0.5800 | [regulation of transcription, DNA-templated](https://www.ebi.ac.uk/QuickGO/term/GO:0006355) | - | [DNA binding](https://www.ebi.ac.uk/QuickGO/term/GO:0003677) |
| U6KP59 | Nascent polypeptide-associated complex subunit beta | 1.6885 | 0.0370 | 1.0757 | 0.7991 | [regulation of transcription, DNA-templated](https://www.ebi.ac.uk/QuickGO/term/GO:0006355) | - | - |
| U6KP66 | Uncharacterized protein | 2.2682 | 0.0408 | 1.5622 | 0.3763 | - | - | - |
| U6KPS4 | Uncharacterized protein | 1.7133 | 0.0336 | 1.3919 | 0.3303 | - | membrane | - |
| U6KQ53 | Proteophosphoglycan ppg1, related | 3.63579 | 0.0274 | 8.9740 | 0.0907 | - | - | - |
| U6KRE5 | Uncharacterized protein | 2.0401 | 0.0262 | 1.8987 | - | - | - | - |
| U6KV40 | Glutamyl-tRNA synthetase, putative | 1.8743 | 0.0458 | 1.7599 | 0.4017 | [glutamyl-tRNA aminoacylation](https://www.ebi.ac.uk/QuickGO/term/GO:0006424) | cytoplasm | [ATP binding](https://www.ebi.ac.uk/QuickGO/term/GO:0005524) [glutamate-tRNA ligase activity](https://www.ebi.ac.uk/QuickGO/term/GO:0004818) [ligase activity](https://www.ebi.ac.uk/QuickGO/term/GO:0016874) |
| U6KX89 | Uncharacterized protein | 1.6806 | 0.0221 | 1.0577 | 0.9055 | intracellular protein transport,vesicle-mediated transport | membrane coat | metal ion binding, phosphoprotein phosphatase activity, structural molecular activity. |
| U6KYX8 | Uncharacterized protein | 2.3390 | 0.0289 | 1.7892 | 0.3461 | - | membrane | - |
| U6L0C1 | 40s ribosomal protein S20, putative ,RP-S20e, RPS20 | 2.2779 | 0.0090 | 1.4586 | 0.4732 | translation | small ribosomal subunit | structural constituent of ribosome |
| U6L620 | Uncharacterized protein | 1.7899 | 0.0231 | 1.6383 | 0.1604 | - | - | - |
| U6L837 | Acetyl-CoA acyltransferase B, putative | 1.7808 | 0.0014 | 1.1772 | 0.6811 | [metabolic process](https://www.ebi.ac.uk/QuickGO/term/GO:0008152) | - | [transferase activity, transferring acyl groups other than amino-acyl groups](https://www.ebi.ac.uk/QuickGO/term/GO:0016747) |
| U6LA11 | Uncharacterized protein | 2.4251 | 0.0206 | 1.6611 | 0.4028 | - | - | - |
| A5JNY6 | Uncharacterized protein | 2.0613 | 0.0381 | 1.1764 | 0.6919 | - | - | - |
| U6KLY6 | Penicillin amidase domain-containing protein, putative | 0.4599 | 0.0198 | 0.0327 | 0.0013 | antibiotic biosynthetic process | - | [hydrolase activity, acting on carbon-nitrogen (but not peptide) bonds, in linear amides](https://www.ebi.ac.uk/QuickGO/term/GO:0016811) |
| U6L0U5 | Subtilisin-like protein, related | 0.4980 | 0.0008 | 0.3000 | 0.0004 | - | membrane | serine type endopeptidase activity |
| U6KN65 | adenosylhomocysteinase | 0.2643 | 0.0254 | 0.3757 | 0.0450 | one carbon metabolic process, S-adenosylhomocysteine catabolic process | - | adenosylhomocysteinase activity, NAD binding |
| U6KZZ1 | uncharacterized protein | 0.2509 | 0.0229 | 0.3085 | 0.0311 | - | - | - |
| U6L225 | uncharacterized protein | 0.3424 | 0.0184 | 0.3362 | 0.0089 | - | - | - |
| H9B8Z9 | 40S ribosomal protein SA | 0.8282 | 0.2547 | 0.2530 | 0.0043 | [ribosomal small subunit assembly](https://www.ebi.ac.uk/QuickGO/term/GO:0000028), [translation](https://www.ebi.ac.uk/QuickGO/term/GO:0006412) | [cytosolic small ribosomal subunit](https://www.ebi.ac.uk/QuickGO/term/GO:0022627) | [structural constituent of ribosome](https://www.ebi.ac.uk/QuickGO/term/GO:0003735) |
| Q3HNM4 | Calmodulin-like domain protein kinase | 0.9125 | 0.6515 | 0.4155 | 0.0396 | - | - | ATP binding,Calcium ion binding, protein kinase activity |
| U6KT65 | Eukaryotic translation initiation factor 5A | 1.0402 | 0.9063 | 0.3352 | 0.0264 | positive regulation of translational elongation, positive regulation of translational termination, translational frameshifting. | - | ribosome binding,translation elongation factor activity,translation initiation factor activity. |
| U6KTF0 | Glutathione peroxidase | 0.4971 | 0.1141 | 0.3883 | 0.0241 | [response to oxidative stress](https://www.ebi.ac.uk/QuickGO/term/GO:0006979) | - | [glutathione peroxidase activity](https://www.ebi.ac.uk/QuickGO/term/GO:0004602) |
| U6KUR9 | ATP-dependent helicase, putaive, putative | 0.8685 | 0.4897 | 0.4516 | 0.0437 | - | - | ATP binding, helicase activity, nucleic acid binding. |
| U6KUT8 | Enoyl-CoA hydratase/isomerase family protein, putative | 0.8901 | 0.4674 | 0.2517 | 0.0232 | [metabolic process](https://www.ebi.ac.uk/QuickGO/term/GO:0008152) | membrane | isomerase activity |
| U6KWM0 | Fructose-1,6-bisphosphatase, putative FBP | 0.8373 | 0.6201 | 0.4978 | 0.0254 | [carbohydrate metabolic process](https://www.ebi.ac.uk/QuickGO/term/GO:0005975) |  | [fructose 1,6-bisphosphate 1-phosphatase activity](https://www.ebi.ac.uk/QuickGO/term/GO:0042132) |
| U6KXG8 | Phosphate carrier protein, putative | 0.5860 | 0.1314 | 0.2121 | 0.0043 | [transport](https://www.ebi.ac.uk/QuickGO/term/GO:0006810) | [membrane](https://www.ebi.ac.uk/QuickGO/term/GO:0016021) |  |
| U6KYQ6 | Uncharacterized protein | 0.7104 | 0.0597 | 0.3470 | 0.0022 | - | - | - |
| U6L2N5 | Eukaryotic translation initiation factor 2 alpha subunit, putative | 0.8656 | 0.3281 | 0.3112 | 0.0141 | - | - | translation initiation factor activity. |
| U6L555 | Seryl-tRNA synthetase, putative SARS | 0.9017 | 0.5536 | 0.2305 | 0.0100 | seryl-tRNA aminoacylation | cytoplasm | ATP binding, serine-tRNA ligase activity |
| U6KK17 | Acyltransferase, putative | 0.4016 | 0.0390 | 0.5338 | 0.1941 | [cellular lipid metabolic process](https://www.ebi.ac.uk/QuickGO/term/GO:0044255) | membrane | [O-acyltransferase activity](https://www.ebi.ac.uk/QuickGO/term/GO:0008374) |
| U6KUP1 | Interferon gamma-inducible protein 30, putative | 0.5889 | 0.0162 | 0.7768 | 0.3204 | - | - | - |
| U6KWG0 | 6-phosphogluconate dehydrogenase, putative | 0.5758 | 0.0391 | 0.1752 | - | - | - | [phosphogluconate dehydrogenase (decarboxylating) activity](https://www.ebi.ac.uk/QuickGO/term/GO:0004616) |
| U6KWN1 | Equisetin synthetase, related | 0.4994 | 0.0297 | 0.1058 | - | - | - | [oxidoreductase activity](https://www.ebi.ac.uk/QuickGO/term/GO:0016491)  [phosphopantetheine binding](https://www.ebi.ac.uk/QuickGO/term/GO:0031177) |
| U6KZ13 | Superoxide dismutase | 0.4483 | 0.0263 | 0.5963 | 0.0761 | - | - | [metal ion binding](https://www.ebi.ac.uk/QuickGO/term/GO:0046872)[superoxide dismutase activity](https://www.ebi.ac.uk/QuickGO/term/GO:0004784) |
| U6L0B6 | Uncharacterized protein | 0.1619 | 0.0084 | 0.9595 | 0.9035 | - | - | - |
| U6L197 | Uncharacterized protein | 0.6370 | 0.0283 | 0.6361 | 0.1930 | - | - | - |
| U6L458 | Alanine dehydrogenase, putative | 0.2459 | 0.0256 | 0.7020 | 0.3486 | [L-alanine catabolic process](https://www.ebi.ac.uk/QuickGO/term/GO:0042853) | - | [alanine dehydrogenase activity](https://www.ebi.ac.uk/QuickGO/term/GO:0000286) |
| U6KY19 | UMP-CMP Kinase.putative | 2.4716 | 0.0486 | 1.945 | 0.1381 | - | Nucleobase-containing compound metabolic process | ATP binding,nucleobase-containing compound kinase activity |
| U6KP87 | - | 2.7389 | 0.0164 | 3.570 | - | - | - | - |
| U6KHL8 | Uncharacterized protein | 1.6111 | 0.0009 | 1.5878 | - | - | - | - |
| H9B9Y7 | Uncharacterized protein | 2.7358 | 0.0246 | 1.4545 | 0.6772 | - | - | - |
| H9B9I2 | Ran-specific GTPase-activating protein,putative | 2.5184 | 0.0036 | 1.2750 | 0.7420 | Intracellular transport | - | - |
| U6KWL2 | Sushi domain-containing protein | 2.1131 | 0.017 | 1.125 | 0.8056 | - | - | - |
| U6KXC3 | Microneme protein Etmic-2 | 1.2555 | 0.7561 | 31.744 | 0.0212 | - | - | - |
| U6L925 | 60s ribosomal protein L13a, putative | 1.2229 | 0.7688 | 2.3559 | 0.0137 | translation | - | Structural constituent of ribosome |
| U6KH97 | RNA binding protein,putative | 1.1921 | 0.8368 | 6.886 | 0.0429 | - | - | RNA Binding |
| U6KWP4 | Tubulin alpha chain | 0.4285 | 0.0003 | 0.5070 | 0.0471 | Microtubule-based process | - | GTPase activity;GTP binding; structural constituent of cytoskeleton |
| U6KW67 | 40S ribosomal protein S17, putative | 0.1645 | 0.0481 | 0.4746 | 0.2626 | translation |  | Structural constituent of ribosome |
| U6KW23 | Peroxisomal biogenesis factor 11 domain-containing protein, putative | 0.4924 | 0.2039 | 0.2583 | 0.0465 | Peroxisome fission | - | - |
| U6KMU3 | Importin alpha. putative | 0.7244 | 0.4555 | 0.2058 | 0.0233 | - | - | - |
| U6KV85 | Uncharacterized protein | 0.8270 | 0.7201 | 0.0294 | 0.0498 | - | - | - |
| U6KSP0 | UBX domain-containing protein, putative | - | - | 0.3753 | 0.0106 | - | - | - |

Biological processes, cellular components and molecular functions were obtained from the Uniprot database. "-" represents not presented.

**Table S10**. The reported drug-target in apicomplexan parasites.

| Parasites | Drug-target gene | Drug | Protein level Regulation  (Drug resistant strains  /Drug sensitive strains ) | Transcriptional level Regulation  (Drug resistant strains  /Drug sensitive strains ) | Copy number | Mutations |
| --- | --- | --- | --- | --- | --- | --- |
| Toxoplasma | dihydrofolate reductase (DHFR) | Pyrimethamine | - | - |  | Yes [38] |
| Plasmodium berghei | Multidrug resistance associated protein (mrp) | Chloroquine | Up[39] | Up [39] |  | Yes |
| Plasmodium falciparum | Multidrug resistance transporter 1 (Pfmdr1) | Chloroquine | - | Up [40] |  | Yes |
| Plasmodium falciparum | K13 | Artemisinin | - | Down-regulated  (early ring stage);  Up-regulated (early trophozoite stage) [41] |  | Yes |
| Plasmodium falciparum | Pfmdr1 | Artemisinin | Up [42] | Up [42] | Increase [42] | Yes |
| Plasmodium falciparum | Chloroquine  resistance transporter (pfcrt) | Chloroquine | No change | No change | No change [43] | Yes |

-: We couldn't find the report on this.

**Supplementary material S1：The calculated method of the four anticoccodial indices.**

ACI = (survival rate + relative weight gain rate) – (lesion value + oocyst value), Growth and survival ratio (GRS) = final body weight/initial body weight, POAA = (GRS in infected-medicated group – GRS in infected-unmedicated group) / (GSR in uninfected-unmedicated group – GRS in infected-unmedicated group) × 100%, RLS = (average lesion score in infected-unmedicated group – average lesion score in infected-medicated group / average lesion score in infected-unmedicated group) × 100%, ROP = (oocyst output in infected-medicated group / oocyst output in infected-unmedicated group) × 100% (Lan et al., 2017).
